# Functional aspects of cerebrospinal fluid drainage through the cribriform plate

**DOI:** 10.1093/braincomms/fcag308

**Published:** 2026-08-11

**Authors:** Nagesh C Shanbhag, Jari Jukkola, Marios Kritsilis, Nicholas B Bèchet, Kelley M Swanberg, Chenchen Liu, Xuanhui Liu, Denys Kovalenko, Na Liu, Emily M Johansson, Anja Meissner, Wondossen Sime, Ramin Massoumi, Graham Louw, René In ‘t Zandt, Iben Lundgaard

**Affiliations:** Department of Experimental Medical Science, Lund University, Lund 221 84, Sweden; Wallenberg Centre for Molecular Medicine, Lund University, Lund 221 84, Sweden; Meditech Foundation, Cali 760031, Colombia; Department of Experimental Medical Science, Lund University, Lund 221 84, Sweden; Wallenberg Centre for Molecular Medicine, Lund University, Lund 221 84, Sweden; Department of Experimental Medical Science, Lund University, Lund 221 84, Sweden; Wallenberg Centre for Molecular Medicine, Lund University, Lund 221 84, Sweden; Department of Experimental Medical Science, Lund University, Lund 221 84, Sweden; Wallenberg Centre for Molecular Medicine, Lund University, Lund 221 84, Sweden; Department of Clinical Sciences, Lund University, Lund 221 84, Sweden; Lund Stem Cell Centre, Lund University, Lund 221 84, Sweden; Department of Cardiothoracic Surgery and Transplantation, Skåne University Hospital, Lund 221 85, Sweden; Department of Experimental Medical Science, Lund University, Lund 221 84, Sweden; Wallenberg Centre for Molecular Medicine, Lund University, Lund 221 84, Sweden; Department of Experimental Medical Science, Lund University, Lund 221 84, Sweden; Wallenberg Centre for Molecular Medicine, Lund University, Lund 221 84, Sweden; Department of Neurology, Tongji Hospital, Tongji Medical College, Huazhong University of Science and Technology, Wuhan 4300 30, China; Department of Experimental Medical Science, Lund University, Lund 221 84, Sweden; Wallenberg Centre for Molecular Medicine, Lund University, Lund 221 84, Sweden; Department of Neurosurgery, Tianjin Medical University General Hospital, Tianjin 3000 52, China; Department of Experimental Medical Science, Lund University, Lund 221 84, Sweden; Wallenberg Centre for Molecular Medicine, Lund University, Lund 221 84, Sweden; Department of Experimental Medical Science, Lund University, Lund 221 84, Sweden; Wallenberg Centre for Molecular Medicine, Lund University, Lund 221 84, Sweden; Department of Experimental Medical Science, Lund University, Lund 221 84, Sweden; Wallenberg Centre for Molecular Medicine, Lund University, Lund 221 84, Sweden; Department of Experimental Medical Science, Lund University, Lund 221 84, Sweden; Wallenberg Centre for Molecular Medicine, Lund University, Lund 221 84, Sweden; Division of Physiology & Vascular Biology, University of Augsburg, Augsburg 86159, Germany; Department of Laboratory Medicine, Division of Translational Cancer Research, Lund University, Lund 223 63, Sweden; Department of Laboratory Medicine, Division of Translational Cancer Research, Lund University, Lund 223 63, Sweden; Division of Clinical Anatomy and Biological Anthropology, Department of Human Biology, Faculty of Health Sciences, University of Cape Town, Cape Town 7935, South Africa; Lund Bioimaging Centre, Centre for Research Infrastructure in Health and Life Sciences, Lund University, Lund 221 84, Sweden; Department of Experimental Medical Science, Lund University, Lund 221 84, Sweden; Wallenberg Centre for Molecular Medicine, Lund University, Lund 221 84, Sweden

**Keywords:** CSF efflux, nasal pathway, cribriform plate

## Abstract

Cerebrospinal fluid (CSF) clearance is essential for maintaining brain homeostasis, yet the contribution of the nasal efflux pathway remains incompletely understood. Here, we combined dynamic contrast-enhanced magnetic resonance imaging, optical imaging, and anatomical studies across mice and pigs to characterize the dynamics, regulation, and conservation of nasal CSF drainage. We found that CSF rapidly exited the brain via the cribriform plate, especially during acute volume shifts, and that this route slowed down in aged mice when a small-molecular-weight tracer was used. By contrast, olfactory sensory neuron ablation and amyloid beta_1-42_ exposure impaired uptake of CSF into the nasal pathway. Notably, signal uptake in superficial cervical lymph nodes appeared distinct from meningeal lymphatic drainage, suggesting functional specialization. Our corroborating findings in pigs further solidify the nasal CSF efflux route as a dynamic, evolutionarily conserved, and potentially targetable pathway with relevance to neuroimmune interactions, aging, and neurodegeneration.

## Teaser

The study by Shanbhag *et al*. reveals that cerebrospinal fluid (CSF) efflux through the cribriform plate is rapid and conserved across mammalian species, modulated by acute CSF volume changes, olfactory neuronal ablation, and amyloid beta_1-42_. The results highlight links to aging, neurodegeneration, and brain-lymphatic function.

## Graphical Abstract

**Graphical Abstract:**
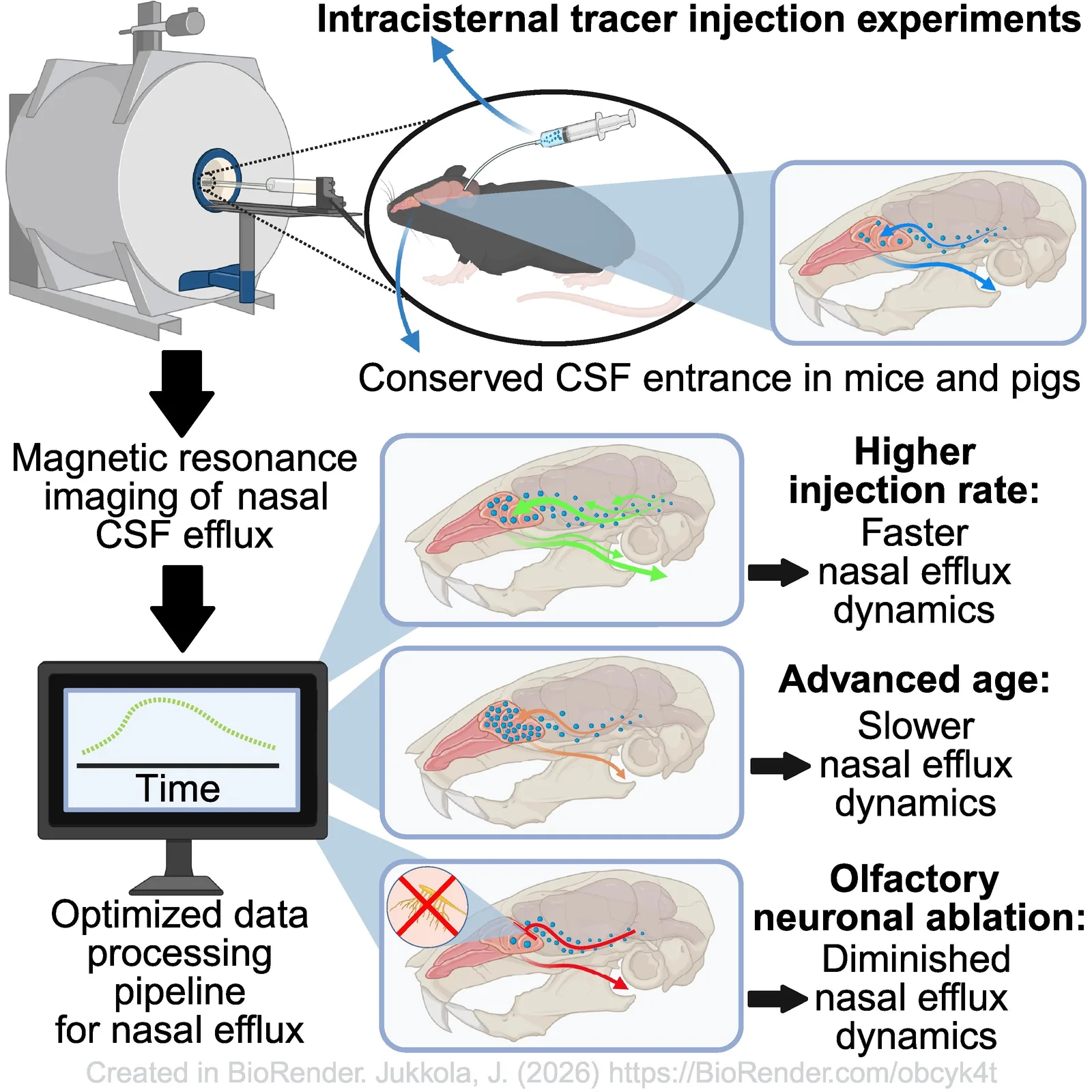

## Introduction

Dysregulated cerebrospinal fluid (CSF) efflux from the brain can have dire consequences for brain integrity, as occurs in hydrocephalus or CSF leaks.^1,2^ Furthermore, stagnation of CSF turnover contributes to the accumulation of toxic proteins and is related to advanced age,^3-7^ which is a risk factor for neurodegenerative diseases such as Alzheimer's disease.^8^ The rediscovered meningeal lymphatic vessels within the dura mater are capable of draining CSF, central nervous system (CNS)-derived antigens, immune cells, and solutes directly to deep cervical lymph nodes, acting as critical routes for CNS immune surveillance.^9-13^ The meningeal lymphatic network can be functionally divided into dorsal and basal compartments, where the basal lymphatics have better access to and drainage capabilities for CSF, with direct downstream connections to nasopharyngeal and cervical lymphatics.^12,13^

Besides these meningeal lymphatic networks, cranial nerves such as optical, trigeminal, and especially the olfactory nerve with its own intra- and extracranial lymphatic hub, are permissible to perineural CSF flow.^14-21^ Quantitative studies in sheep have estimated that at least 40–50% of CSF drains to extracranial lymphatic vessels,^22,23^ but the exact CSF fractions draining through the aforementioned dorsal and basal meningeal lymphatics and along optical and trigeminal nerve are unknown. Contrasting these efflux routes, other studies in sheep have shown that during homeostatic intracranial pressure (ICP) levels, the cribriform plate acts as the primary CSF efflux site, and that its experimental blockade drastically increases ICP and CSF outflow resistance.^24-26^ It is therefore not surprising that the nasal efflux pathway remains one of the most robustly supported and evolutionarily conserved efflux sites for CSF, which drains through the cribriform plate along olfactory nerve bundles and towards the lymphatics of the nasal turbinates (NTs) and nasopharyngeal lymphatic channels, before reaching the cervical lymphatic systems.^13,27-30^

While being an important route for CSF efflux, most prior studies of the lymphatic network at the cribriform region have relied on *ex vivo* or histological approaches,^31-34^ limiting insight into its functional dynamics *in vivo*. Moreover, comparative studies across species evaluating CSF efflux capacity and regulation via this nasal efflux pathway are scarce, leaving a significant gap in our understanding of the physiological importance of CSF clearance, particularly under varying proteinopathies like Alzheimer’s and Parkinson’s disease.^35-39^ To gain new insights into the functional dynamics of nasal CSF efflux, we employed dynamic contrast-enhanced magnetic resonance imaging (DCE-MRI), as well as *in vivo* and *ex vivo* stereomicroscopic approaches. Furthermore, we performed corroborating investigations on the nasal efflux pathway in pigs.

## Materials and methods

### Mice

Adult male wild-type C57BL/6J (8–16 week and 25–26-month-old) mice purchased from Charles River/Janvier were housed at the animal facility of Lund University in standard laboratory conditions (shared room and cages with four to five animals per cage), with a 12h:12 h light-dark cycle with *ad libitum* access to normal chow and water. After acclimatization of at least 5 days, a total of 110 mice was used at the animal facility. Different treatment conditions were performed, when possible, in alternating order within each experiment day.

### Pigs

Fifty to sixty kilograms male Landrace pigs (*Sus scrofa domesticus*) purchased from an approved supplier were housed at the animal facility of Lund University for 7 days prior to the experiments at an ambient temperature of 22–25°C and 30–70% humidity on a 12 h light-dark cycle. The pigs had *ad libitum* access to drinking water and food. All experiments were performed at the animal facility.

All investigations in mice and pigs conform to the CODEX guidelines by the Swedish Research Council, Directive 2010/63/EU of the European Parliament on the protection of animals used for scientific purposes and Regulation EU 2019/1010 on the alignment of reporting guidelines and were conducted in accordance with the ethical guidelines approved by the Malmö-Lund ethical Committee on Animal Research (5.8.18-08269/2019, 5.8.18-20240/2021, 5.8.18-04510/2024 and 5.8.18-05527/2019). A total of five pigs were used in the experiments.

### CSF tracer preparation

The gadolinium-based contrast agent (GBCA, gadoterate meglumine, Dotarem®, 0.75 kDa Guerbet LLC, Villepinte, France) for DCE-MRI experiments was diluted in 0.9% NaCl to a final concentration of 12.5 mM and stored at +4°C for later use. For fluoroscopy experiments, Alexa Fluor™ 647 conjugated bovine serum albumin (BSA-647, 67 kDa, A34785, Invitrogen/Thermo Fisher Scientific, CA, USA), was diluted in artificial CSF (aCSF, mM: 126 NaCl, 2.5 KCl, 1.25 NaH_2_PO_4_, 2 MgCl_2_, 2 CaCl_2_, 10 glucose, 26 NaHCO_3_, pH 7.4) to a concentration of 1–2% w/v. HiLyte™ Fluor 555-labeled beta amyloid (1–42) (Aβ_1-42_- HiLyte™555, AS-60480-01, Anaspec, CA, USA) was prepared by adding 10 μL of 1% NH_4_OH and 90 μL of aCSF to 0.1 mg of lyophilized tracer powder. Lumiprobe-Cy7.1 (1.2 kDa, carboxylic acid, 26390, Lumiprobe) was diluted in aCSF to a concentration of 0.05% w/v for IVIS-CT.

### Intracisternal tracer infusion in mice

Mice were anesthetized with an intraperitoneal (i.p.) injection of KX (100 mg/kg and 20 mg/kg, respectively). Mice were placed on a heated small animal physiological monitoring device connected to a head fixation frame (Harvard Apparatus). Upon unresponsiveness to a toe pinch, the skin overlying the neck muscles was excised and the muscles retracted to reveal the cisterna magna (CM) as previously described.^40^ For the DCE-MRI experiments, a ∼30 G MR-compatible copper needle (outer diameter 0.32 mm; Nippon Tokushukan MFG Co. Ltd., Tokyo, Japan) connected to a 25 μl Hamilton syringe via PE10 tubing was used and the cannula was secured using super glue (LOCTITE®). An infusion speed of 4 μl/min, 1 μl/min or 0.4 μl/min was used to inject 10 μl of the GBCA via a remote-controlled infusion pump (Harvard Apparatus PHD 2000, MA, USA) during the scan.

For light microscopy *in vivo* imaging, the skin covering the dorsal skull and nasal turbinate region was incised and removed. CM injection was then carried out using a stainless-steel 30 G dental needle (Sopira® Carpule®) connected to a 100 μl Hamilton syringe via PE10 tubing, and tracer was injected using a single infusion syringe pump (LEGATO® 100 Syringe pump, KD Scientific, MA, USA). For the injection rate and volume challenge, mice received either 10 μl of 1% BSA-647 with an injection rate of 4 μl/min or 1 μl/min, or alternatively, 5 μl of 2% BSA-647 with an injection rate of 0.2 μl/min. The slow group had half the volume and 2x tracer concentration to enable full injection within the total 40-min experimental timeline, while injecting the same amount of fluorophore. For the co-injection experiment, mice received a total of 8 μl of tracer solution made up of a mixture of 5 μl of BSA-647 and 3 μl of either aCSF or Aβ_1-42_- HiLyte™555 with an injection rate of 1 μl/min. For the *ex vivo* tissue clearing, mice received 10 μl of 1% BSA-647 with an injection rate of 1 μl/min.

For *in vivo* imaging system combined with computed tomography (IVIS-CT) (IVIS® Spectrum CT, Revivity, Perkin Elmer, MA, USA-CT), mice under KX anesthesia were first shaved around the skin overlying dorsal and ventral surfaces of the head and neck using Veet® cream. This was performed to avoid interference from laser light traversing through hair pigments during imaging. Upon securing the intracisternal cannula as described above, mice were positioned on a 37°C heated imaging chamber over a transillumination platform of the imaging system. A near-infrared CSF tracer Cy7.5 was infused over 10 min (10 μl, 1 μl/min), followed by a 30-min circulation time.

### Intracisternal tracer infusion in pigs

First, five Landrace pigs underwent overnight fasting with access to drinking water. On the day of surgery, the animal was anesthetized with a mixture of ketamine, midazolam, and fentanyl (KMF) as described previously.^41^ Heart rate, blood pressure, body temperature, and pCO_2_ were continuously recorded during the entire procedure. The skin overlying the back of the head and neck was resected, and the underlying muscles were severed and retracted to expose the CM. An 18 G cannula was inserted into the CM and fixed in place with super glue. Intracisternal injection of BSA-647 was carried out manually using a 1 ml syringe catheter at a rate of 100 μl/min, for a total of 800 μl, in anesthetized pigs. Tracer was allowed to circulate for 2 h prior to sacrifice with intravenous pentobarbital. Brain, olfactory bulbs, the NTs, and dorsal and skull base dura mater were carefully dissected and post-fixed in 4% paraformaldehyde (PFA), before being used for further processing.

For all intracisternal experiments, data from mice and pigs with either clear leakage from the injection site, minimal, or non-existent tracer presence in downstream tissues were disregarded.

### Dynamic contrast-enhanced magnetic resonance imaging (DCE-MRI)

Magnetic resonance (MR) scanning was performed on a 9.4 Tesla preclinical scanner (BioSpec Avance III 94/30; Bruker, Ettlingen, Germany; magnet from Agilent, Santa Clara, USA) with actively shielded gradients (maximum 670 mT/m; rise time 130 μs) and integrated shims (BGA 12S HP, Bruker, BioSpin, Ettlingen, Germany). Hardware was controlled via ParaVision 7.0.0 software. Imaging was performed using a transmit-receive ^1^H quadrature cryocoil (CryoProbe^TM^; Bruker, Ettlingen, Germany).

After CM cannula implantation, animals underwent DCE-MRI. The animal was carefully positioned on the imaging bed, and the head was fixed using customized ear bars attached to a head holder. Before placement into the scanner bore, anesthetized animals received a supplementary dose of ketamine (50 mg/kg, i.p.). After insertion into the bore, body temperature was stabilized at 36.5–37.0°C using a circulating warm-water system (Lauda RC6, Lauda-Königshofen, Germany), while both temperature and breathing rate were monitored with a physiological monitor (SA Instruments, NY, USA). After this, manual tuning and matching, automated adjustments of transmit frequency and power, receive gain, global shims, and mouse positioning relative to the magnet isocenter were confirmed and adjusted using a three-orthogonal-slice *T_1_*-weighted localizer. Dynamic contrast-enhanced imaging series consisted of whole-head 3D fast low-angle shot (FLASH) acquisitions (*T_E_* 1.5 ms, *T_R_* 10 ms, *α* 10°, field of view FOV 150 × 200 × 100 mm^3^, 0.1-mm isotropic voxels, acquisition time *T_A_* 2.5 min, number of time series acquisition volumes *N* = 26). Following two baseline acquisitions (∼5 min), GBCA was injected into CM via a remote-controlled infusion pump at a rate of 1 μl/min, except as specified for the injection rate groups in the injection rate challenge experiment. FLASH acquisitions were divided into two blocks of *N* = 6 and *N* = 20, respectively, to enable early experimental termination in the case of visible injection failure. The time it took to start the second scan was 13 s and this delay was accounted for in the timeline analysis. After *N* = two pre-injection baseline acquisitions, GBCA was infused and allowed to circulate for ∼63 min. Thereafter, the animal was euthanized by cervical dislocation. Every experiment that demonstrated significant net enhancement outside the injection site and/or lack of net enhancement within brain parenchyma, suggestive of substantial tracer leakage or otherwise failed injection, were terminated after the first block of six scans and excluded from further analysis.

### Nasal turbinate segmentation and DCE-MRI signal processing

Two time series of 3D-FLASH volumes acquired (*N* = 6 + 20) were exported in DICOM format and imported into MICE Toolkit, MTK (v. 2002.4.9, NONPI Medical AB, Umeå, Sweden). The volumes from the two separate acquisition blocks (*N* = 6 and *N* = 20) were combined in one time series and associated with time vectors representing volume acquisition midpoints, including the 13-second delay caused by the time needed to start the second batch of 20 time points. Briefly, volumes from two separate acquisition blocks (*N* = 6 and *N* = 20) were combined in series and associated with time vectors representing volume acquisition midpoints. Volumes were motion-registered against the first timepoint by interface with elastix^42,43^ and averaged over time to serve as a reference for blinded (group identity encryption by another researcher) manual masking of the NT or the olfactory bulb region of interest (ROI). Signals considered for summation over masked ROIs were subtracted from pre-injection baseline (average of the first two volumes), denoised by time-domain principal component analysis (six components), and filtered to exclude all voxels that did not represent at least an enhancement signal that was three times the standard deviation of the background noise. NT ROI was manually defined first against predominantly transverse, followed by coronal, averaged anatomical reference scan slices as the entire nasal region, as previously described.^44^ Otsu multi-step thresholding^45^ was applied to these whole-nasal region masks to yield NT tissue masks, which were in turn multiplied with the aforementioned contrast filter to yield enhancing NT masks, over which signals were summed for further statistical analysis.

### IVIS-CT

During *in vivo* imaging using IVIS-CT, relevant anatomical regions of interest were selected (two overlying MCA-supplied territories, olfactory bulbs, nasal and cervical regions) to acquire 3D images of tracer distribution. These regions were selected on the 2D image captured as a baseline in order to visualize tissue auto-fluorescence prior to administration of the fluorescent dye. Image acquisition was carried out before and during the tracer infusion and the 30-min circulation time. Epi-fluorescent 2D and trans-fluorescent 3D Image acquisition was carried out using 745 nm excitation (Ex 745 nm) and 800 nm emission (Em 800 nm) spectrum during the experiment. Living image® software (version 4.5.5) was used for image acquisition and data analysis (PerkinElmer, MA, USA, v.4.7.3).

### Pharmacological ablation of olfactory sensory neurons

Upon anesthesia with isoflurane, 10 μl of 10% v/w zinc-sulfate (ZnSO_4_, AC389802500 ThermoFisher Scientific/Acros organics) in sterile saline or vehicle (sterile saline) was administered onto each of the nostrils using a 10-μl pipette in an alternating droplet instillation method (2 × 2.5 μl dose/nostril, 5-min break between rounds) onto both nostrils. Following the treatment, the animal was kept in prone position to allow for excess fluid to exit the nasal cavity. Monitoring for general wellbeing and body weight was carried out after treatment. The mice underwent either DCE-MRI on Day 7 or Day 28 after the treatment, or alternatively, were sacrificed for scanning electron microscopy (SEM) or histological staining 7 or 28 days after the treatment.

### Histopathological staining in mice

For morphological assessment of intranasally administered ZnSO_4_ solution on olfactory epithelium, mice were decapitated either on Day 7 or Day 28, and whole mouse heads were fixed in 4% PFA solution for 48 h. Thereafter, the tissue was decalcified with 10% ethylenediaminetetraacetic acid (EDTA, 8040, Roth) solution (w/v in MilliQ water) for 12 days at 4°C. The solution was changed every 48 h. Finally, the whole head was embedded in optimal cutting temperature (OCT) compound (Tissue-Tek®, Sakura, ref. 4583) and cut into 15 μm sections using a cryotome and mounted on slides. Hematoxylin and eosin staining were performed. Sections were imaged under a light microscope (Olympus CX 33) and a dissection microscope (Nikon SMZ 745T).

### In vivo transcranial fluorescence microscopy and ex vivo imaging in mice

For *in vivo* transcranial fluorescence imaging, after the CM cannula was glued to the skull, the mouse was positioned under a Nikon SMZ25 stereomicroscope with a Plan Apo 0.5x objective (0.08 NA) equipped with an Andor Zyla 4.2 Plus sCMOS camera (Mag-0.75x, Zoom-1.5x). The excitation wavelength was 635 nm using a CoolLED pE4000 LED illumination and the emission filter used was a quadruple bandpass filter. CSF circulation and efflux to the NT were imaged *in vivo*, at 1-min intervals for 30 min following the end of injection. From the same animals, after overnight fixation in 4% PFA, the skull cap containing the dorsal dura (exposure time 80 ms), the superficial cervical lymph nodes (SCLNs) (exposure time 30 ms) and deep cervical lymph nodes (DCLNs) (exposure time 50 ms) were imaged. The dorsal dura was then carefully dissected from the skull cap, mounted on a slide and imaged with a Nikon Ti2 wide-field fluorescence microscope at 4× magnification (Plan Apo 4x, 0.2 NA, exposure time 500 ms). For the quantitative analysis of tracer distribution in different tissues, images were imported into Fiji,^46^ manual ROIs were drawn after blinding by group identity encryption by another researcher, and the mean fluorescence intensities were calculated.

### *Ex vivo* imaging in pigs

Following the *in vivo* injections in pigs, whole brains, the NTs, olfactory bulbs, and whole dorsal and ventral dura mater were imaged similarly under a stereomicroscope. Furthermore, dorsal dura mater and the NTs, decalcified with 10% EDTA, were embedded in OCT compound sectioned with a cryostat into 20 μm-thick frozen sections, and mounted for gross and microscopic evaluation of tracer distribution using stereomicroscopy and wide-field imaging, respectively.

### Optical tissue clearing, light-sheet imaging, and analysis

After tracer circulation, mice were transcardially perfused with 1× PBS, followed by 4% PFA. The whole head and spine of each mouse were carefully dissected and postfixed overnight at 4°C in 4% PFA. The samples were then decalcified in 10% EDTA for 24 h. Samples were washed in 1× PBS, and the immunolabeling-enabled three-dimensional imaging of solvent-cleared organs (iDISCO+) protocol was carried out as previously described.^47,48^ Briefly, tissues were dehydrated in increasing methanol/H_2_O series (20%, 40%, 60%, 80%, 100%, 1 h each and 100% overnight), delipidated with methanol/dichloromethane (33%/66% for 3 h), and pure dichloromethane (two times, 15 min each), and optically cleared with ethyl cinnamate (ECi) for at least 1 week prior to imaging. Following optical clearing, we performed light-sheet imaging of the samples using a LaVision UltraMicroscope Blaze light-sheet microscope (Miltenyi Biotec, Bergisch Gladbach, Germany) controlled by ImSpectorPro software (Miltenyi Biotec, Bergisch Gladbach, Germany). All samples were imaged while immersed in ECi, with the 1.1× objective (Miltenyi Biotec MI PLAN 1.1×, extra magnification 1×, effective magnification 1.1/0.1 NA) in the transverse orientation at a step size of 4 μm. The dual-sided three-sheet illumination was used, along with the mosaic acquisition setting to obtain consecutive z-stacks with 10% overlap. The 16-bit TIFF images acquired from light-sheet imaging were imported to Arivis Vision4D v3.5 (Zeiss Arivis, Rostock, Germany) for 3D reconstruction. Tiled images of the head-spine samples were manually aligned in the XY axes based on visual inspection, using the Tile Sorter function of Arivis Vision4D, and representative 3D images and videos of the reconstructed samples were then exported.

### Scanning electron microscopy of nasal turbinate region

On the seventh day from ZnSO_4_ or saline intranasal administration, mice were sacrificed by cervical dislocation under brief isoflurane anesthesia. Excess soft tissue around the nose was excised, including the lower jaw. To collect the entire nose without damaging the structure, a razor was used to separate part of the olfactory bulbs and the entire nose from the head. Tissue was immersed in 1:1 ice-cold solution of 10% EDTA and 2× fixative (0.1 M Sorensen´s phosphate buffer pH 7.4, 2% formaldehyde and 2% glutaraldehyde) overnight at 4°C. Samples were washed 3 × 5 min with 1× PBS and immersed in 10% EDTA for six more days (7 days total in EDTA). The samples were washed again for 3 × 5 min with ice-cold 1× PBS followed by immersion in Sorensen´s buffer. The samples were then dehydrated in a graded series of ethanol (50%, 70%, 80%, 90%, and twice in 100% for 1 h each) followed by critical point drying and mounting. After the samples were dry and glued to a holder, a razor was used to separate the tip of the nose from the rest of the sample at ∼7.5 mm from the tip. The exposed mounted tissue was then coated with a mixture of platinum and palladium and imaged (after blinding by group identity encryption by another researcher) with scanning electron microscope (Jeol JSM-7800F).

### Statistical analysis

All statistics were calculated in R v. 4.3.1 (R Project for Statistical Computing, Vienna, Austria). Summed signal intensities for imaging time series, preprocessed as described above, as well as integral and slope (backward differences) thereof, were fit by generalized linear mixed models with independent variables group (injection rate, age, zinc sulfate treatment, or Aβ_1-42_), time point (for DCE-MRI, midpoint of 2.5-min FLASH acquisition blocks 1-26), and their interaction with random individual intercepts; analysis of variance (ANOVA) on general linear mixed models was then used to assess model coefficient statistical significance. Post hoc pairwise comparisons were achieved by *t*-test. *P*-values were corrected for multiple comparisons within each linear model by the Benjamini–Hochberg method.^49^ Standalone between-group comparisons of single variables were conducted as one-way ANOVA (=Student’s *t*-test for two groups), with post hoc Tukey’s correction. Linear model residuals were assessed for normality by Shapiro–Wilk test,^50^ and Kruskal–Wallis test followed by Wilcoxon rank-sum test was conducted for group comparisons of single variables in the case of Shapiro–Wilk test significance (*P* < 0.05). Descriptive statistics are reported as means ± standard error of the mean (shaded area around data points in time series and whiskers in bar charts). Uncorrected *α* was defined at 0.05. In all the groups, the experimental unit is one mouse or pig. Visual representations of precalculated statistics were done using GraphPad Prism (version 10).

## Results

### Higher cisterna magna injection rate increases CSF solute efflux along the nasal route

Hydrocephalus, in which ICP is elevated due to impaired CSF flow, currently lacks acute experimental models that avoid obstruction or major confounds in CSF dynamics. In contrast to this pathological state, physiologically relevant manipulations such as downward head tilt or blood pressure lowering have been shown to acutely elevate ICP and enhance CSF efflux in anesthetized mice.^51,52^ To establish an acute and controllable model of elevated CSF pressure for studying efflux through the cribriform plate, we infused 10 µl of gadolinium-based contrast agent (GBCA, 12.5 mM Dotarem®) into the cisterna magna (CM) under ketamine-xylazine (KX, 100 mg/kg and 20 mg/kg) anesthesia at controlled rates of 0.4 µl/min (low), 1 µl/min (moderate), and 4 µl/min (high). Using DCE-MRI and our optimized data processing pipeline for NT signal enhancement,^53^ we could visualize signal enhancement caused by our tracer in the NTs over time (Fig. 1A-C). Faster injection rates led to earlier and more pronounced signal enhancement peaks, while the slowest rate resulted in delayed and diminished signal dynamics (Fig. 1D). The time to maximum enhancement varied significantly with infusion rate, with the 4 μl/min group showing the fastest signal rise (Fig. 1E). We then analyzed the magnitude of tracer enhancement change. Corresponding slope analyses revealed steeper slopes before and after maximum enhancement in this group (Fig. 1F-H), suggesting that higher infusion rates generated more robust CSF pressure gradients, which in turn accelerated clearance through the cribriform plate and led to significantly increased total enhancement accumulation (Fig. 1I and J). Interestingly, despite marked differences in early tracer kinetics, signal enhancement in the NTs reached similar levels across all groups by 60 min, underscoring the nasal route’s capacity to act as a pressure valve that can dissipate acute intracranial volumetric shifts.

**Figure 1:**
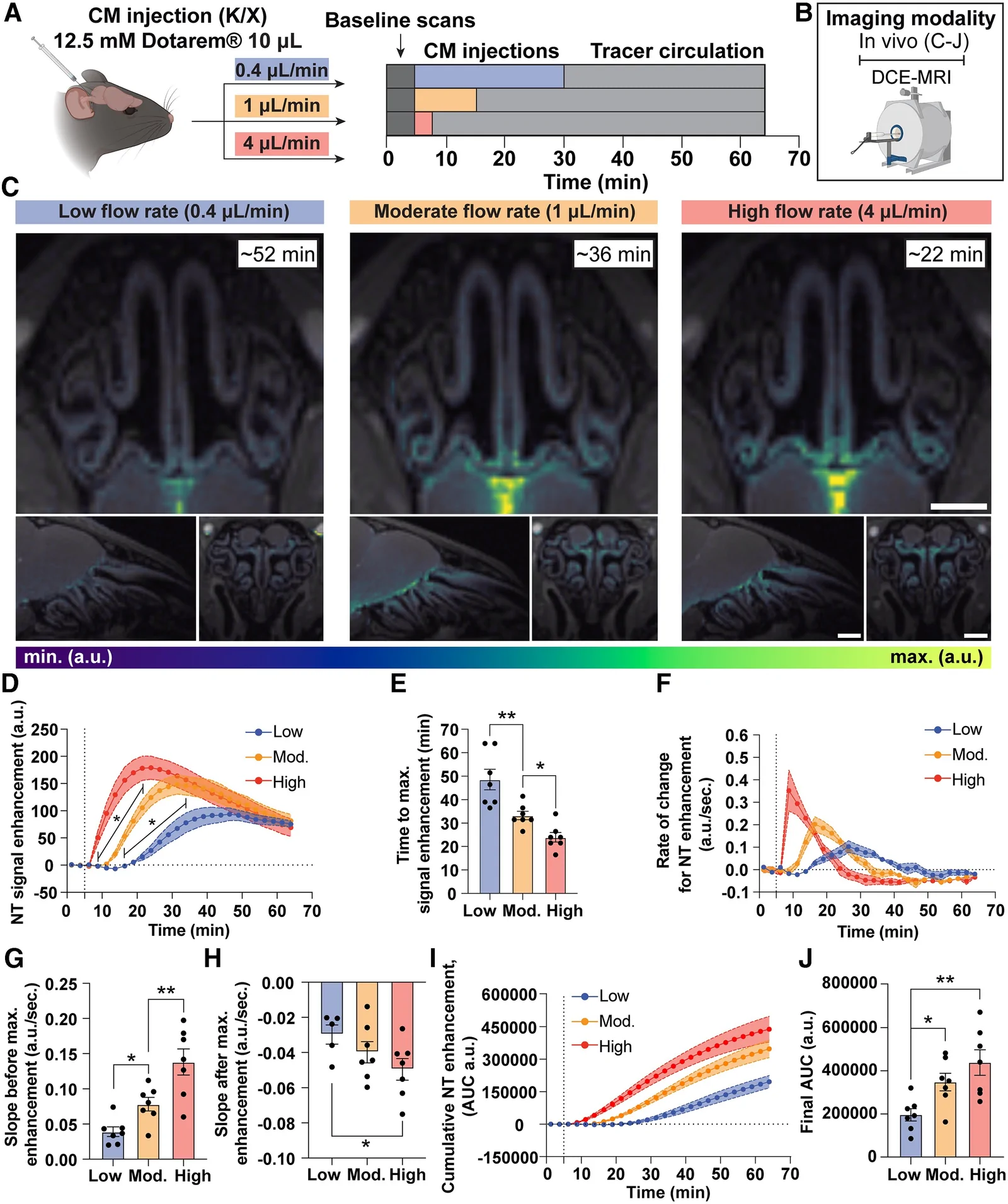
**The effect of cisterna magna (CM) injection rate on cerebrospinal fluid tracer kinetics during dynamic contrast-enhanced magnetic resonance imaging (DCE-MRI).** (**A**) After setting DCE-MRI parameters, a baseline is measured before tracer is injected either at 0.4 μl/min (low), 1 μl/min (moderate = Mod.), or 4 μl/min (high) into CM under ketamine-xylazine (KX) anesthesia. (**B**) 9.4 T preclinical MR rodent scanner, equipped with a cryocoil, is used for imaging the signal enhancement. **(C)** Representative images of the nasal turbinates (NTs) at axial (*top*), sagittal (*bottom left*), and coronal (*bottom right*) views at peak signal enhancement (arbitrary units, a.u.) timepoints for each respective mouse depicted. Scale bars: 1 mm. **(D)** Time—enhancement curves of NT signal enhancement during the experiment (dashed gray line indicates the start of tracer injection). **(E)** Time to maximum NT signal enhancement. **(F)** Rate of change for NT signal enhancement. Multiple timepoints are statistically significant (not shown for visual clarity). **(G, H)** Slopes from experiment start to maximum signal enhancement, and from maximum signal enhancement to experiment end. For the low-group, two mice had their maximum signal enhancement as their final datapoint and were excluded from this analysis. **(I)** Area under curve (AUC, cumulative NT enhancement). Most timepoints are statistically significant (not shown for visual clarity). **(J)** Final AUC from the last timepoint of (**I**). For all-time series data, one-way ANOVA on mixed models (*P* < 0.05 for all), followed by post hoc *t*-test for pairwise comparisons for each timepoint with Benjamini–Hochberg correction, were used, where * = *P* < 0.05. For non-time series group comparisons, one-way ANOVA (*P* < 0.05 for all but slope after max. enhancement), followed by post hoc pairwise comparisons for each injection rate group with Tukey’s correction, were used, where * = *P* < 0.05, ** = *P* < 0.01. Spheres and shaded areas in time series represent the mean value for each group from all mice per group, and the standard error of the mean, respectively. Spheres and whiskers in bar charts represent each mouse and the standard error of the mean, respectively. *n* = Seven male mice per group. Created in BioRender. Jukkola, J. (2026) https://BioRender.com/gnxg653.

While the MR cryocoil enabled high signal-to-noise and precise visualization of the NT signal enhancement, reliable assessment of downstream lymphatic clearance was restricted in mice kept in a prone position, as these regions are too far away from the coil. To visualize this clearance route, we employed IVIS-CT following intracisternal injection using 1 μl/min injection rate (Supplementary Fig. 1). Real-time imaging revealed anterior movement of tracer from the cisterna magna toward the olfactory bulbs and the NTs, consistent with pressure-assisted solute clearance. In the cervical region, tracer signal localized predominantly to the superficial cervical lymph nodes (SCLNs), with secondary localization in the deep cervical lymph nodes (DCLNs). These findings support a model in which nasal CSF efflux under KX anesthesia facilitates rapid drainage into the peripheral lymphatic system via established olfactory-lymphatic conduits.

### Superficial cervical lymphatic pathway connects to the nasal efflux pathway and disseminates acute CSF volume shifts

To further characterize the anatomical and functional connections between the NTs and downstream lymphatic drainage, we injected fluorescent tracer BSA-647 (10 μl, 1 μl/min) into CM under KX anesthesia and allowed circulation for 5, 30, or 180 min. Following transcardial perfusion, the head, neck, and spinal column were harvested as an intact unit and optically cleared using the iDISCO + protocol^47,48^ (Fig. 2A-C). Light-sheet imaging confirmed rapid tracer movement through the nasal pathway within 5 min of injection, alongside near-simultaneous accumulation within the spinal canal, including uptake in the dorsal root ganglia of the lumbar region (Fig. 2C). In the cervical region, DCLNs consistently exhibited greater tracer signal than the SCLNs. Being in line with previous findings,^33^ high-resolution imaging revealed distinct lymphatic afferents: SCLNs appeared directly connected to the NTs, while DCLNs were in close proximity to the cervical spine (Fig. 2D and E), suggesting two separate efflux routes: one nasal and anterior, and one spinal and posterior, each with a distinct spatial relationship to the CSF compartment.

**Figure 2:**
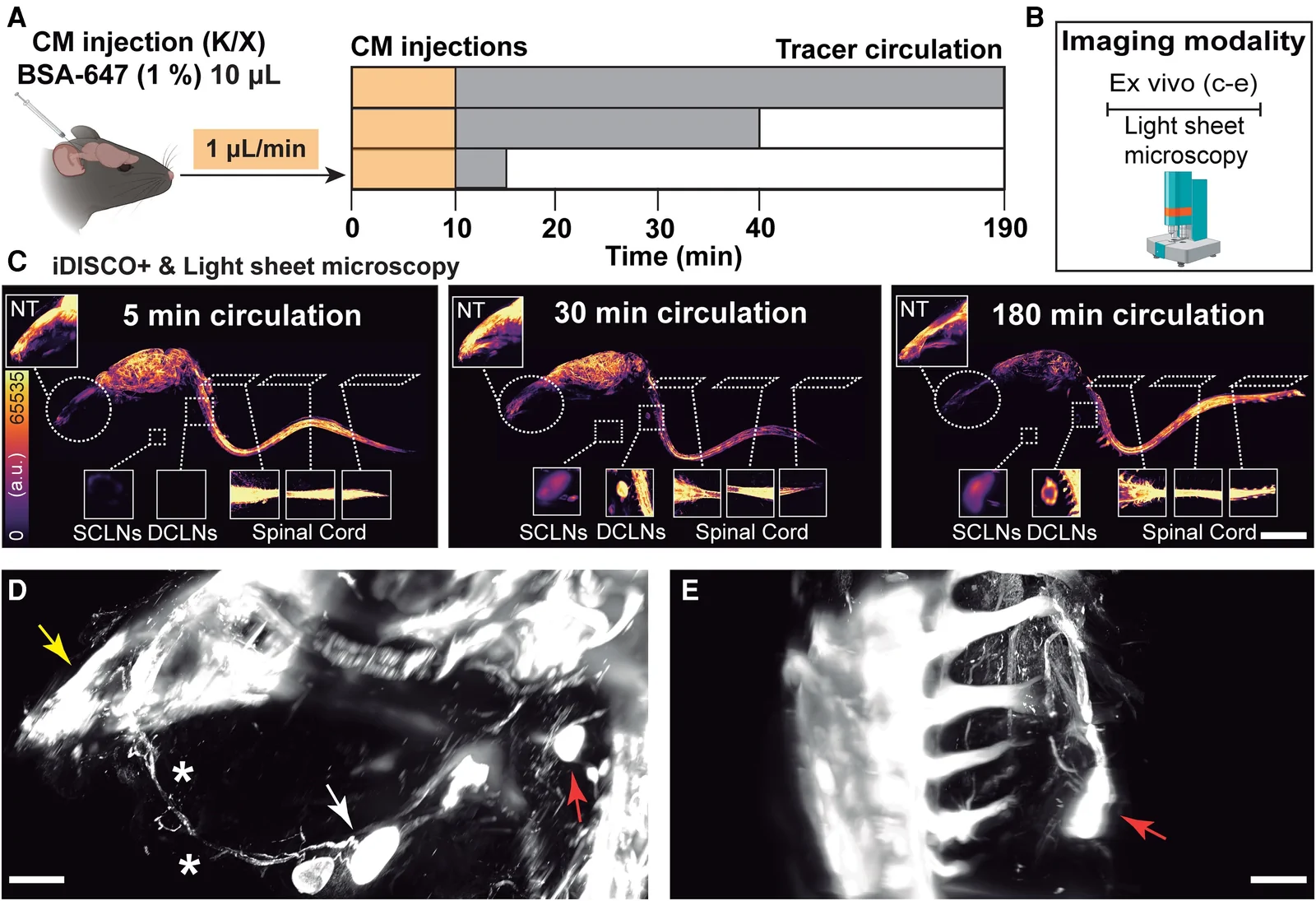
**Nasal cerebrospinal fluid (CSF) uptake and drainage to superficial cervical lymph nodes form a major efflux pathway in mice. (A, B)**. Experimental timeline for the cisterna magna (CM) injection and the imaging modality utilized after CSF tracer circulation under ketamine-xylazine (KX) anesthesia. **(C)** Immunolabeling-enabled three-dimensional imaging of solvent-cleared organs (iDISCO+) was used to reveal tracer kinetics (arbitrary units, a.u.) in intact head-spine samples. Insets of representative images show tracer presence in the nasal turbinates (NTs), superficial (SCLNs) and deep (DCLNs) cervical lymph nodes and different parts of the spinal cord after 5, 30, or 180 min of circulation, respectively. Scale bar: 5 mm. **(D, E)** Representative 3D reconstructions of cleared head-spine samples of mice following 30 min of tracer circulation, with focus either on the lymphatic connection (white asterisks) between the SCLNs (white arrow) and the NTs (yellow arrow) or DCLNs (red arrow) and the spinal cord. Scale bars: 1 mm. *n* = Three mice per time point. Created in BioRender. Jukkola, J. (2026) https://BioRender.com/3rcqz9f.

To evaluate how these efflux pathways respond to acute fluid volumetric challenges, we repeated CM injections of BSA-647 using three different infusion rates, but adjusted tracer concentration and volume to maintain a constant total tracer mass (Fig. 3A). To align tracer distribution with peak nasal signal observed via DCE-MRI, the total experimental duration was limited to 40 min. The groups were as follows: 5 μl of 2% BSA-647 at 0.2 μl/min (low), 10 μl of 1% at 1 μl/min (moderate = mod.), and 10 μl of 1% at 4 μL/min (high). At 40 min, tracer fluorescence was markedly elevated across the dorsal cranial surface, including the NTs, in the high-group (Fig. 3B-D). Among the draining lymph nodes, the SCLNs—positioned downstream of nasal efflux—showed significantly higher signal in the moderate and high—groups compared to the low-group, (Fig. 3E). By contrast, DCLN signal did not significantly differ among injection groups (Fig. 3F), suggesting their drainage may be less sensitive to acute CSF volume changes. Finally, increased tracer accumulation in the skull cap and dorsal dura only occurred in the high -group and was very modest (Fig. 3G and H), further supporting a limited role for dural-associated clearance under high flow conditions. These findings indicate that during acute CSF volume increase, efflux via the nasal route, culminating in the SCLNs, acts as a responsive and pressure-sensitive clearance mechanism. Surprisingly, the drainage to the DCLNs was not enhanced by moderate or high injection rates, suggesting a passive drainage that perhaps serves immune function more than relieving CFS pressure.

**Figure 3:**
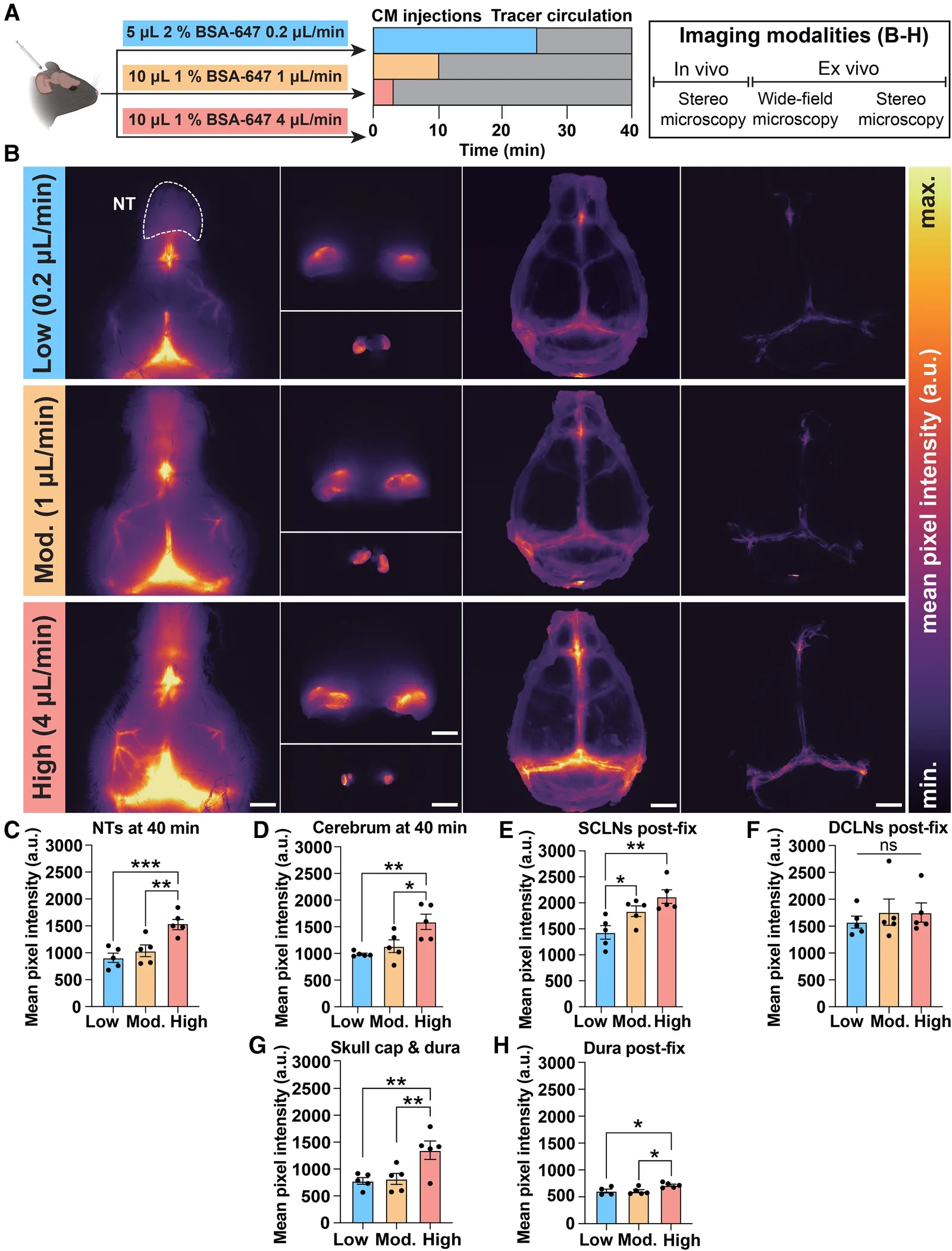
**Different cisterna magna (CM) injection rates followed by *in vivo* and *ex vivo* microscopy. (A)** Experimental timeline for the CM injection rate experiment and imaging modalities for *in vivo* and *ex vivo* outcomes. **(B)** Representative images of the whole dorsal skull surface and the nasal turbinates (NTs) at 40 min, and *ex vivo* imaging of superficial cervical lymph nodes (SCLNs) depicted on top of deep cervical lymph nodes (DCLNs), isolated skullcap with dura, and isolated dura (wide-field microscopy). Scale bars: 2 mm. **(C-H)** Quantification of mean pixel intensities (arbitrary units, a.u) for the NTs *in vivo* at 40 min (**C**), cerebrum *in vivo* at 40 min (**D**), SCLNs (**E**), DCLNs (**F**), skullcap with dura (**G**), and isolated dura mater (**H**). For all regions and tissues other than DCLNs, one-way ANOVA (*P* < 0.05 for all), followed by post hoc pairwise comparisons for each injection rate group with Tukey’s correction, was used. For DCLNs, Kruskal–Wallis test (*P* > 0.05) followed by post hoc Wilcoxon rank-sum test with Benjamini–Hochberg corrected *P*-values were used. For all results, * = *P* < 0.05, ** = *P* < 0.01, *** = *P* < 0.001, ns = not significant. *n* = Five mice per group. Spheres and whiskers in bar charts represent each mouse and standard error of the mean, respectively. Created in BioRender. Jukkola, J. (2026) https://BioRender.com/sivrvh0.

### Nasal CSF efflux dynamics slow down with aging and are impaired by Aβ_1-42_

Given the utility of *in vivo* DCE-MRI in capturing acute CSF dynamics in the NTs, we next investigated the impact of aging on nasal efflux. As 1 µl/min is commonly used in the field^44,54-56^ and only transiently affects ICP,^57^ it was used for all subsequent experiments (Fig. 4A). Under KX anesthesia, young (2–4 months) and aged (2 years) mice underwent CM tracer injection of GBCA (10 µl), followed by DCE-MRI. Surprisingly, the overall volume of enhancing voxels within the NTs did not differ significantly between young and aged mice (Fig. 4B). However, aging led to significantly slower enhancement dynamics in the NTs, as evidenced by significantly diminished influx phase, longer time to max. enhancement, and lingering signal enhancement (Fig. 4C and D). Furthermore, aged mice had a significantly elevated rate of change in tracer signal at several time points compared to younger mice (Fig. 4E), although the signal increase before the maximum enhancement and the decline thereafter were similar between groups (Fig. 4F and G). Despite these kinetic differences, cumulative signal (area under the curve) and final enhancement levels were comparable between age groups (Fig. 4H and I). Even after normalization of signal by enhancing voxel count, aged mice maintained significantly different signal enhancement and rate of change dynamics (Supplementary Fig. 2A, D) but had no marked differences in slopes or total signal accumulation dynamics (Supplementary Fig. 2B, C, E, F). However, there was no difference in absolute signal enhancement in the olfactory bulbs (Supplementary Fig. 2G, H). These results suggest that aging does not impair CSF passage to olfactory bulbs, but there seems to be higher resistance across the cribriform plate and diminished clearance in the periphery. Having established that aging does not dramatically impair nasal CSF intake but might slow down further drainage, we next investigated whether amyloid beta (Aβ_1-42_)—its accumulation being a hallmark of Alzheimer’s disease—might compromise this clearance pathway. To examine this, we co-injected fluorescent BSA-647 with either Aβ_1-42_-HiLyte™555 or vehicle (artificial CSF) into the CM (8 µl, 1 µl/min) and monitored nasal clearance using *in vivo* transcranial imaging for 40 min (Fig. 4J and K). Compared to controls, mice receiving Aβ_1-42_ exhibited a significant reduction in BSA-647 signal in the NTs at later time points (Fig. 4L and M). Analysis of tracer signal in the SCLNs confirmed this effect, with reduced BSA-647 accumulation in the amyloid group (Fig. 4N and O). We further analyzed the data by measuring mean pixel intensities at the last timepoint from olfactory bulb (OB) region and NT region (Supplementary Fig. 2I, J, K) and saw that while NT mean intensities were significantly different between the groups (Supplementary Fig. 2J), there was no difference in the OB mean intensities (Supplementary Fig. 2K). Finally, we investigated the dynamics between mean pixel intensities of NT and OB regions. The ratios between mean intensities in NT versus OB showed no difference (Supplementary Fig. 2L), indicating that the reduced passage of CSF tracer to NTs was driven by intracranial differences caused by Aβ_1-42_.

**Figure 4:**
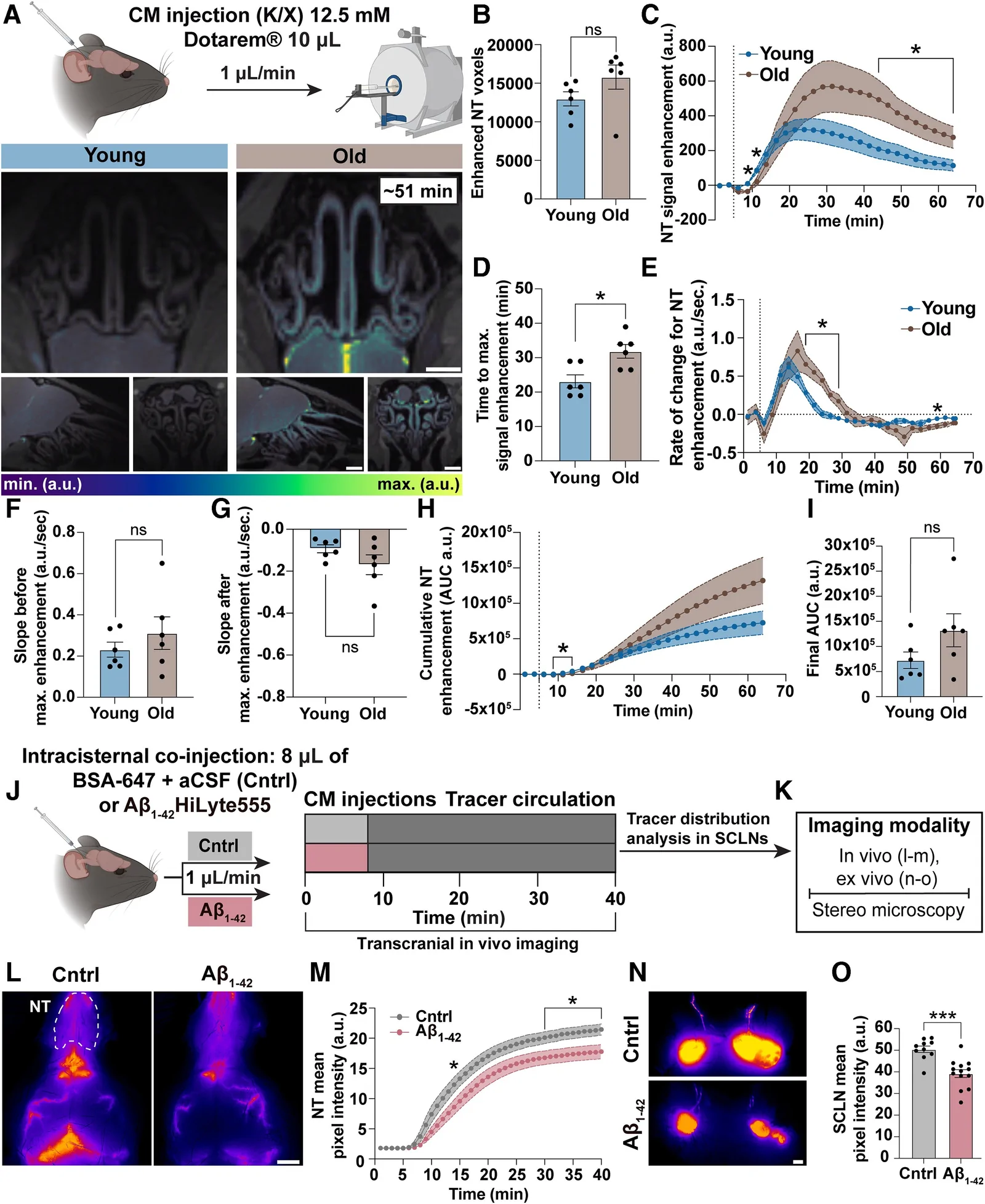
**Nasal cerebrospinal fluid (CSF) efflux dynamics across the cribriform plate are slowed down by aging, whereas amyloid beta (Aβ_1-42_) diminishes nasal efflux and drainage to superficial cervical lymph nodes (SCLNs). (A)** Representative images of the nasal turbinate (NT) signal enhancement (arbitrary units, a.u) at axial (top), sagittal (bottom left) and coronal (bottom right) views at ∼51 min of the experiment with cisterna magna injection (CM) of CSF tracer under ketamine-xylazine (KX) anesthesia. Scale bars: 1 mm. **(B)** Number of enhanced NT voxels in young and old mice. **(C)** Time—enhancement curves of NT signal enhancement. Dashed gray line indicates the start of tracer injection (12.5 mM Dotarem®, 10 μl, 1 μl/min). **(D)** Time to maximum NT signal enhancement. **(E)** Rate of change. **(F, G)** Slopes from experiment start to maximum signal enhancement, and from maximum signal enhancement to experiment end. **(H, I)** Area under curve (AUC, cumulative NT enhancement), with final AUC from the last timepoint of (**H**). *n* = Six mice per group. **(J, K)** Experimental timeline of the co-injection experiment. BSA-647 was co-injected with either artificial CSF (aCSF) in controls (Cntrl) or Aβ_1-42_-HiLyte™555 with imaging modality for both *in vivo* and *ex vivo* outcomes. **(L)** Representative images of fluorescent BSA-647 distribution on the whole dorsal brain surface and NT (dashed line) in vivo. Scale bar: 2 mm. **(M)** Time—intensity curves of the mean fluorescence intensity at the NT. **(N)** Representative *ex vivo* images of SCLNs. Scale bar: 1 mm. **(O)** Quantification of mean fluorescence intensity of SCLNs. *n* = 10 (Cntrl) and *n* = 13 (Aβ_1-42_-HiLyte™555). For all-time series data, ANOVA on mixed models (*P* < 0.05 for NT signal enhancement, cumulative NT enhancement, and NT mean pixel intensity, but *P* > 0.05 for rate of change), followed by post hoc *t*-test for pairwise comparisons for each timepoint with Benjamini–Hochberg correction, were used, where * = *P* < 0.05. For non-time series group comparisons in (**B**), the Wilcoxon rank-sum test was used, whereas for other group comparisons, Student’s *t*-test was used, where * = *P* < 0.05, *** = *P* < 0.001, ns = not significant. Spheres and shaded areas in time series represent the mean value for each group from all mice per group, and the standard error of the mean, respectively. Spheres and whiskers in bar charts represent each mouse and the standard error of the mean, respectively. Created in BioRender. Jukkola, J. (2026) https://BioRender.com/8vnpmwm.

Our findings indicate that while aging does not impair CSF passage through the cribriform plate, acute amyloid burden in the CSF can disrupt this efflux pathway and downstream lymphatic drainage, having direct implications for glymphatic dysfunction and neurodegeneration. Although the administered amount of Aβ_1-42_ represents a relatively high bolus dose, similar effects may also arise from lower levels of exposure sustained over an extended period.

### Ablation of olfactory sensory neurons disrupts CSF efflux through the nasal pathway

Olfactory sensory neurons form a direct anatomical pathway for CSF to exit the cranial compartment via the cribriform plate into the nasal submucosa. We therefore examined how the ablation of this neuronal layer affects nasal CSF efflux. Intranasal zinc sulfate (ZnSO_4_) administration is a well-established method to ablate olfactory sensory neurons and their projections through the cribriform plate.^34,58-60^ As such, this approach provides a useful paradigm to test whether disruption of olfactory structures alters CSF transit across the cribriform plate and its subsequent distribution within nasal tissues during *in vivo* measurements.

Intranasal instillation of 10% ZnSO_4_ caused progressive degeneration of nasal structures. By Day 28, histological analysis showed expected notable submucosal tissue degeneration (Supplementary Fig. 3A-C), whereas by Day 7 post-treatment, SEM confirmed ablation of olfactory sensory neurons in the main NTs (Supplementary Fig. 3D). To assess functional consequences for CSF efflux, we performed CM injections with GBCA followed by DCE-MRI at these two timepoints (Figs 5A and B and 6A and B) in ZnSO_4_-treated and saline-treated mice. On Day 7, ZnSO_4_-treated mice showed significantly reduced signal enhancement in the NTs compared to controls (Fig. 5C), indicating impaired CSF transit through the cribriform plate. However, dynamic parameters including time to maximum enhancement, pre- and post-maximum slopes, and total accumulation did not differ significantly (Fig. 5D-G). Notably, anatomical scans revealed swollen NTs of ZnSO_4_-treated mice on Day 7 (Fig. 5H), with a significant increase in nasal tissue voxel counts (Fig. 5I), suggesting inflammation-related tissue expansion.

**Figure 5:**
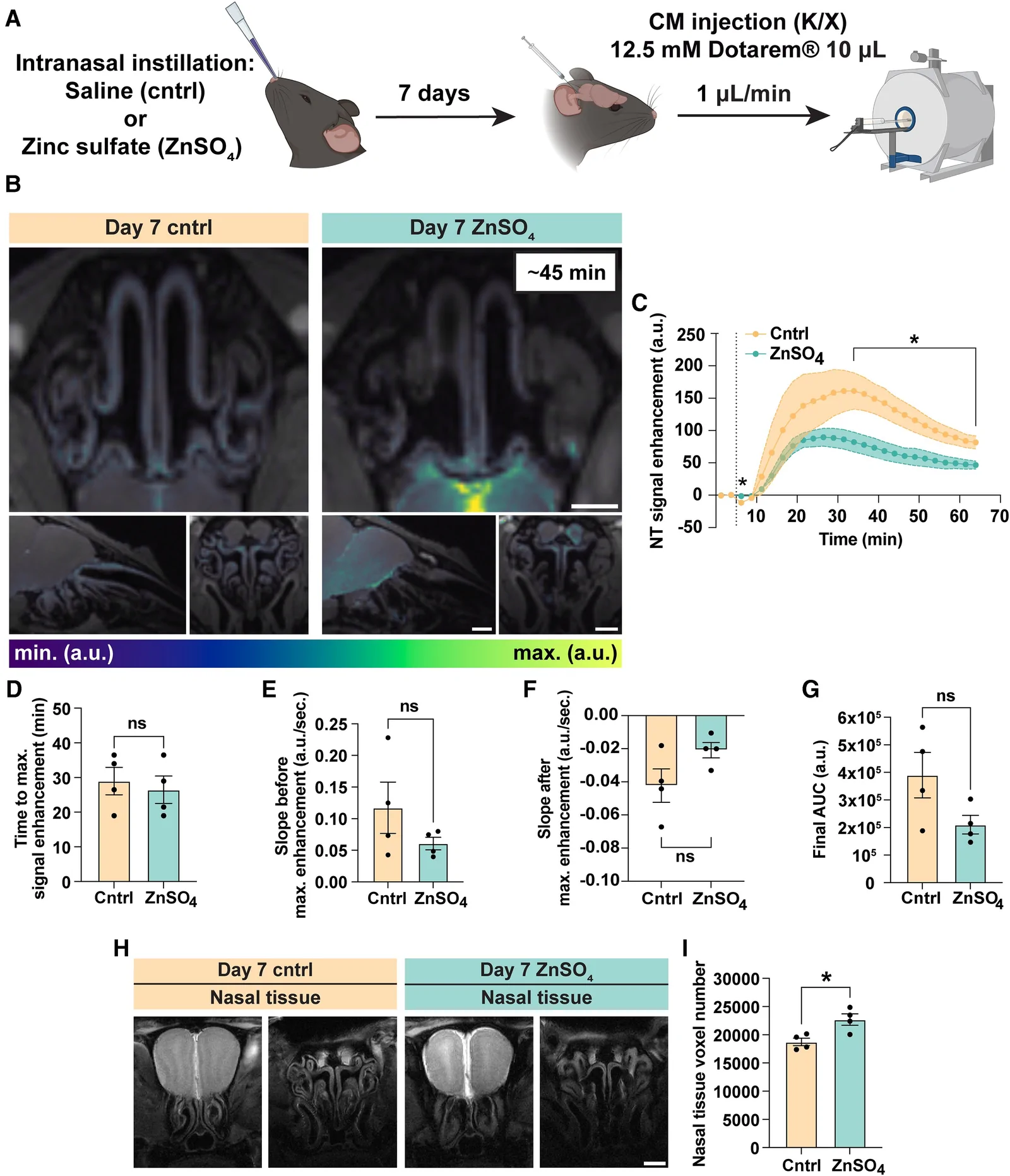
**Olfactory sensory neuron ablation diminishes nasal cerebrospinal fluid efflux on day seven after intranasal administration.** (**A**) Experimental timeline of intranasal zinc sulfate (ZnSO_4_) or saline (in controls, Cntrl) administration and cisterna magna (CM) injection during dynamic contrast-enhanced magnetic resonance imaging (DCE-MRI) under ketamine-xylazine (KX) anesthesia. **(B)** Representative images of the nasal turbinate (NT) signal enhancement (arbitrary units, a.u) at axial (*top*), sagittal (*bottom left*) and coronal (*bottom right*) views on Day 7 at ∼45 min of the experiment. **(C-G)** Characteristics of tracer efflux to the NTs at 7 days after the treatment, including the time—enhancement curves (dashed gray line indicates the start of tracer injection) (**C**), the time to maximum NT signal enhancement (**D**), the slopes before and after the maximum signal enhancement (E, **F**) and final area under curve (AUC) values from the last timepoint (**G**). **(H, I)** Representative coronal images of the nasal area and nasal tissue voxel numbers on Day 7. For all-time series data, ANOVA on mixed models (*P* < 0.05), followed by post hoc *t*-test for pairwise comparisons for each timepoint with Benjamini–Hochberg correction, were used, where * = *P* < 0.05. For non-time series group comparisons, Student’s *t*-test were used, where * = *P* < 0.05, ** = *P* < 0.01, *** = *P* < 0.001, ns = not significant. Spheres and shaded areas in time series represent the mean value for each group from all mice per group, and the standard error of the mean, respectively. Spheres and whiskers in bar charts represent each mouse and standard error of the mean, respectively. *n* = Four mice per group. Scale bars: 1 mm. Created in BioRender. Jukkola, J. (2026) https://BioRender.com/qd38c87.

**Figure 6:**
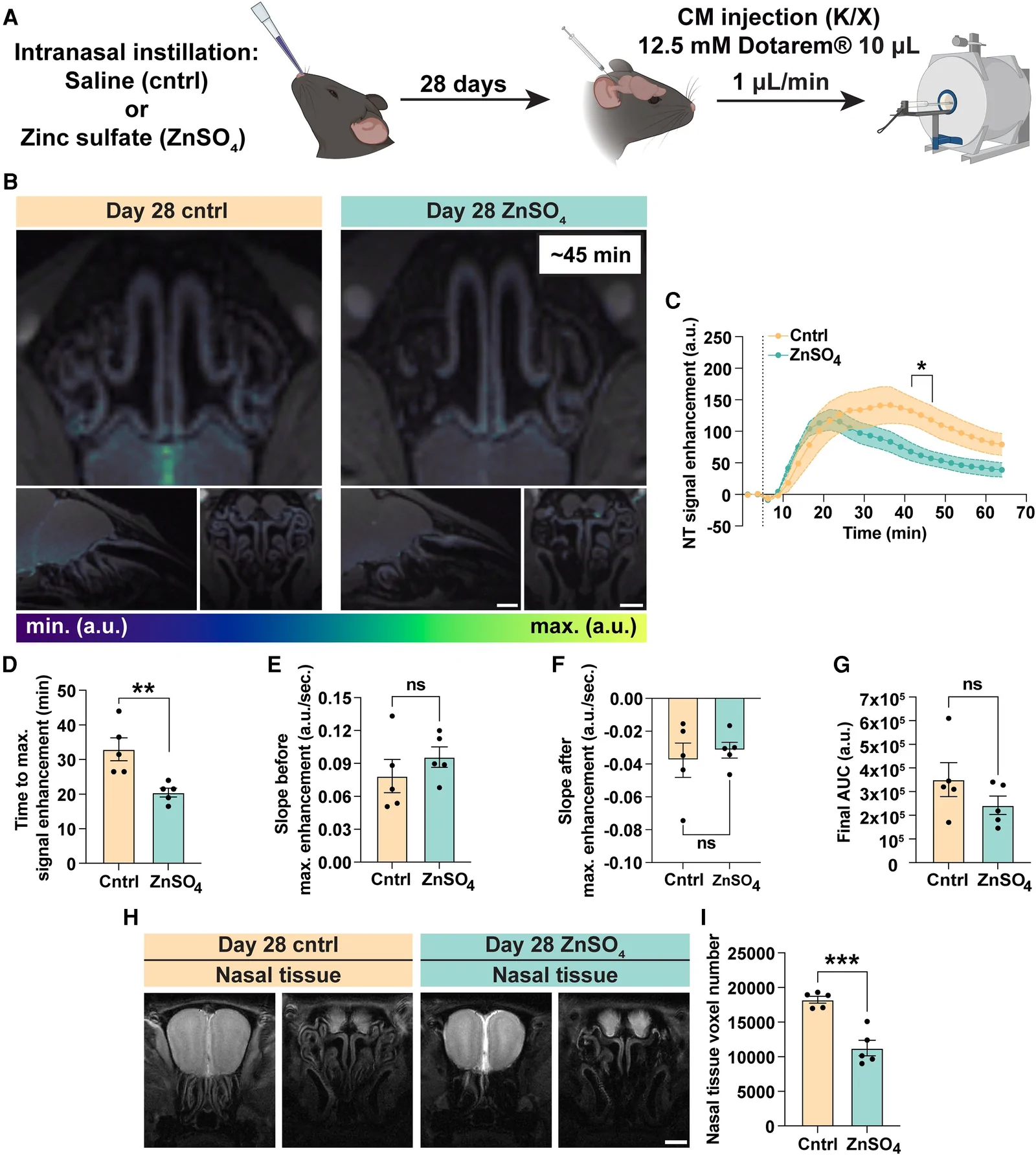
**Olfactory sensory neuron ablation diminishes nasal cerebrospinal fluid efflux on Day 28 after intranasal administration. (A)** Experimental timeline of intranasal zinc sulfate (ZnSO_4_) or saline (in controls, Cntrl) administration and cisterna magna (CM) injection during dynamic contrast-enhanced magnetic resonance imaging (DCE-MRI) under ketamine-xylazine (KX) anesthesia**. (B)** Representative images of the nasal turbinate (NT) signal enhancement (arbitrary units, a.u) at axial (*top*), sagittal (*bottom left*), and coronal (*bottom right*) views on Day 28 at ∼45 min of the experiment. **(C-G)** Characteristics of tracer efflux to the NTs at 28 days after the treatment, including the time—enhancement curves (dashed gray line indicates the start of tracer injection) (**C**), the time to maximum NT signal enhancement (**D**), the slopes before and after the maximum signal enhancement (E, F) and final area under curve (AUC) values from the last timepoint (**G**). **(H, I)** Representative coronal images of the nasal area and nasal tissue voxel numbers on Day 28. For all-time series data, ANOVA on mixed models (*P* < 0.05), followed by post hoc *t*-test for pairwise comparisons for each timepoint with Benjamini–Hochberg correction, were used, where * = *P* < 0.05. For non-time series group comparisons, Student’s *t*-test were used, where * = *P* < 0.05, ** = *P* < 0.01, *** = *P* < 0.001, ns = not significant. Spheres and shaded areas in time series represent the mean value for each group from all mice per group, and the standard error of the mean, respectively. Spheres and whiskers in bar charts represent each mouse and the standard error of the mean, respectively. *n* = Five mice per group. Scale bars: 1 mm. Created in BioRender. Jukkola, J. (2026) https://BioRender.com/w3fz1kl.

By Day 28, signal enhancement in the NTs remained reduced in the ZnSO_4_ group, though the difference was less pronounced (Fig. 6C). Unexpectedly, these mice reached peak enhancement faster than controls (Fig. 6D), but rates of change and overall signal accumulation remained comparable (Fig. 6E-G). Anatomical scans at this later timepoint showed an opposite pattern to Day 7: ZnSO_4_-treated mice exhibited marked tissue loss and reduced nasal voxel counts (Fig. 6H and I), likely reflecting sustained degeneration following the initial injury. To account for changes in enhancing tissue volume, we normalized enhancement signals by the number of enhancing voxels. At Day 7, despite a significant reduction in the number of enhancing voxels (Supplementary Fig. 3E), normalized signal intensity and enhancement dynamics were not significantly different between ZnSO_4_ and control mice (Supplementary Fig. 3F, G). At Day 28, enhancing voxel counts remained similarly reduced (Supplementary Fig. 3H), but normalized enhancement and rate of change were paradoxically increased in the ZnSO_4_ group (Supplementary Fig. 3I, J), suggesting altered fluid distribution or compensation. These findings underscore the essential role of olfactory sensory neurons and nasal submucosa in sustaining CSF efflux through the cribriform route, with their loss leading to early edema and later structural degeneration that progressively alters this clearance pathway.

### Cross-species conservation of nasal CSF efflux pathways in pig

To further explore the translational relevance of nasal CSF efflux, we investigated tracer movement in a pig model, where BSA-647 was injected into the CM *in vivo*, followed by a 2-h circulation period and *ex vivo* tissue imaging (Fig. 7A and B). Dissection and imaging of the brain, olfactory bulbs, the NTs, and dorsal and ventral dura revealed robust tracer presence in regions associated with CSF efflux, including the nasal mucosa and olfactory bulbs, but notable absence from the carotid sheath (Fig. 7C-N). These findings suggest that, like rodents, pigs exhibit rostral CSF clearance into the nasal submucosa, strongly supporting the evolutionary conservation of a rostral CSF clearance pathway through the cribriform plate to the nasal mucosa.

**Figure 7:**
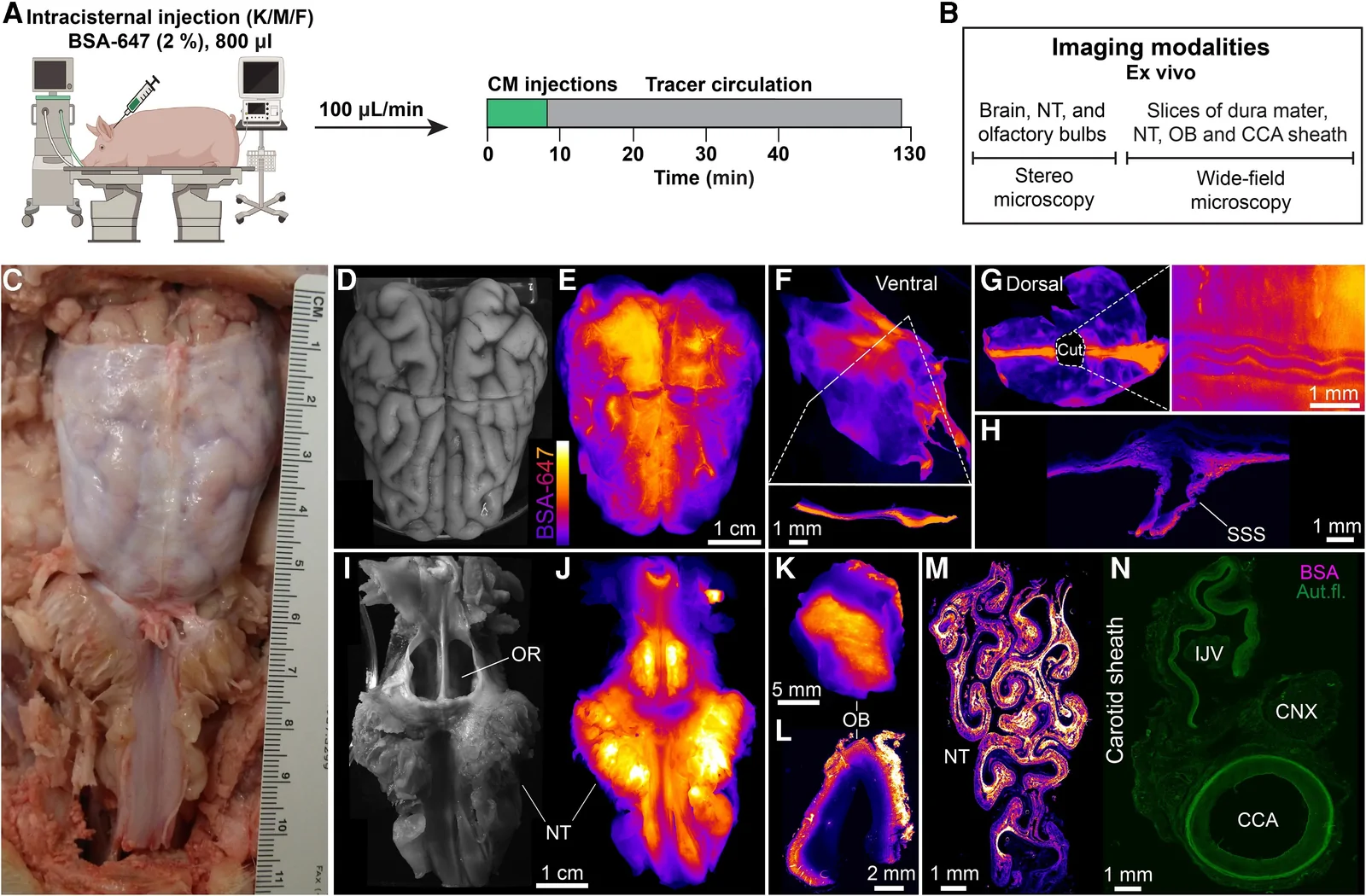
**Pig *in vivo* cisterna magna (CM) injections suggest similarities in nasal efflux function. (A)** Experimental timeline for *in vivo* CM injection of BSA-647 in ketamine-midazolam-fentanyl (K/M/F) anesthetized pigs. **(B)**  *Ex vivo* imaging modalities for brain, dura mater, nasal turbinates (NTs), olfactory bulbs (OB), and common carotid artery (CCA). **(C)** Pig brain and the NTs, with intact dorsal dura covering the cerebrum. **(D, E)** BSA-647 signal in the brain. **(F, G)** Both ventral and dorsal dura mater. **(H)** Insert from dorsal dura showing superior sagittal sinus (SSS). **(I, J)** NTs with olfactory recess (OR). **(K, L, M)** Sections of an OB and NT. **(N)** Carotid sheath containing CCA, internal jugular vein (IJV), and vagus nerve (10th cranial nerve, CNX). BSA-647 (BSA) in purple, autofluorescence (aut.fl.) in green. *n* = Five pigs. Created in BioRender. Jukkola, J. (2026) https://BioRender.com/3lqdcxk.

## Discussion

This study identifies nasal turbinate efflux as a quantifiable and conserved route for CSF clearance across species using a multimodal imaging strategy in live mice and pigs. Using DCE-MRI, we developed a novel method to isolate turbinate signal enhancement *in vivo*, validated against IVIS-CT, which is affected by CSF volumetric changes, aging, and olfactory neuronal damage. Together, these findings characterize the functionality of the nasal pathway, further validating the pathway’s significance in CSF homeostasis that is conserved across mammalian species.

Our results align with prior demonstrations that the olfactory region constitutes a major extracranial exit point for CSF. Classic tracer studies in rodents showed rapid delivery of intraventricular and cisternal tracers to the nasal mucosa and cervical lymphatics.^29,61^ The demonstration of tracer enhancement in the superficial cervical lymph nodes strengthens the interpretation that the nasal pathway provides a functional conduit into systemic lymphatics, agreeing with previous studies.^28,34,51,61^ Our findings suggest that the nasal CSF pathway serves as a compensatory outlet during transient increases in CSF flow dynamics, linking central fluid dynamics with peripheral lymphatic clearance. While previous anatomical findings and our results have refined our understanding of CSF efflux, key uncertainties persist, particularly regarding the distinction between passive pressure-driven flow and active, biologically regulated transport, something that cannot be resolved *ex vivo*. Despite this limitation, our BSA-647 tracer studies show clear CSF tracer presence in the nasal turbinates of mice and pigs. Furthermore, just like in mice, the tracer was observed in the dural meninges following injection in pigs but not in the carotid sheath.

Our results show that relatively more tracer flows to the nasal compartment under differing flow rates when compared to dorsal dura and the lymphatics housed therein, which earlier work has shown to be involved in brain clearance and efflux.^52,62^ However, due to their relatively smaller anatomical structures in general, meningeal lymphatics may be more prone to drainage dysfunction, as seen related to migraine, severely increased ICP, and traumatic brain injury.^63-65^ Therefore, efflux through the cribriform plate or via other, albeit less accessible cranial nerves, could perhaps be considered a more compliant route for CSF efflux as suggested by earlier studies,^24-26^ whereas the meningeal lymphatics could have more pronounced immunological responsibilities.

The delayed and prolonged levels of NT enhancement in aged mice observed in our study are consistent with reports of impaired glymphatic transport and solute clearance in aging brains.^66-68^ CSF production rate is diminished in aged mice,^69^ possibly leading to an attenuated CSF flow toward the cribriform plate as well. The slightly delayed enhancement in aged mice during early timepoints of our signal enhancement and longer time to max. enhancement support this proposition, partly agreeing with recent studies.^70,71^ While the results of these studies show diminished CSF efflux to the NTs and are not entirely recapitulated by our findings regarding the age-related signal enhancement persistence in the NTs, key methodological differences separate our experiments from previous studies: First, the tracers used by these studies are manyfold larger than the <1 kDa GBCA we utilized. While larger tracers are indeed more common outside the context of radiography or DCE-MRI, mouse and human CSF-tracking studies have been conducted using smaller or similar-sized tracers to GBCA used in our study,^72-78^ allowing potentially greater translational power for our findings. Contrasting these small tracers, Decker *et al*.^71^ used larger (17 kDa) GBCA to limit potential intraparenchymal diffusion efficiency after injecting into the lateral ventricle. Considering the size-dependent perivascular access of very large GBCAs,^79^ the passage through the cribriform plate is still possible but perhaps weaker for these larger tracers, as cribriform plate foramina closure is linked with aging in humans.^80^ Smaller tracers could therefore better reflect the potential movement of CSF in the system. Second, unlike in our experiments, the previous studies used isoflurane during the experiments, which heavily affects CSF flow and efflux dynamics.^72^ Third, our optimized data processing pipeline allows greater resolution in defining the anatomical structures within the NTs, resulting in a more robust signal detection. And fourthly, and perhaps most importantly, our injection volume and rate were higher (10 μl, 1 μl/min) than in these studies (0.1 μl/min for 60 min, or 5 μl, 0.5 μl/min, respectively), enabling a more pressure-based CSF flow dynamics in our experiments. Considering these differences, our data may reflect a rerouting of CSF flow from glymphatic influx to efflux across the cribriform plate, which is combined with age-related decreases in meningeal and downstream extracranial lymphatic drainage efficiency.^12,13,28,61^ These factors would explain the delayed and prolonged signal enhancement dynamics in the NTs of aged mice.

The reduced tracer penetrance into NTs and drainage into SCLNs after co-injection with Aβ_1-42_ indicates that this pathway is vulnerable to acute exposure to high levels of Aβ_1-42_, suggesting that chronic low-level exposure may similarly disrupt CSF efflux through the cribriform plate. Supporting this, studies in APP/PS1 mice show Aβ_1-42_ deposition within perineural spaces of olfactory nerve bundles and lymphatic vessels crossing the cribriform plate, impairing efflux.^81^ Chemical ablation of olfactory sensory neurons with zinc sulfate also markedly reduced tracer efflux, consistent with prior work showing degeneration of the olfactory system.^34^ This degeneration, initially observed as edematous nasal tissue and later as reduced tissue volume, likely contributes to impaired CSF drainage to the nasal mucosa. Together, these findings suggest that aging, Aβ_1-42_ aggregation, and neuronal damage can compromise nasal clearance and potentially exacerbate neurodegeneration.

There is currently no interspecies consensus on nasal CSF efflux under varying physiological conditions, although prior large-animal and human studies support its translational relevance.^29,82-85^ While our data from pigs cannot capture *in vivo* dynamics, they support a conserved efflux route in higher mammals. *In vivo* assessment remains challenging, but recent studies in non-human primates (*Macaca fascicularis*) using intracisternal tracers demonstrated drainage via periorbital, nasal, hard palate, and superficial cervical lymphatics, consistent with pathways observed in mice.^28^ Moreover, recent dynamic PET and long-term MRI studies show that intracranial CSF tracers cross the cribriform plate in humans.^83,84^ Similarly, an MR study found that after intrathecal gadobutrol injection, nasal compartments reached peak signal intensities within 10 h, while signal in the parasagittal dura continued increasing beyond 72 h.^86^

Clinically, nasal clearance may provide a measurable biomarker of glymphatic function, accessible via MRI or optical methods, and may be particularly informative in disorders where impaired protein clearance is central, such as Alzheimer’s disease, Parkinson’s disease, and idiopathic intracranial hypertension.^39,66,83,87^ At the same time, nasal CSF efflux represents a potential vulnerability for pathogen entry, as shown in viral invasion through the olfactory route in mice and humans,^88,89^ suggesting a dual role in health and disease.

Several limitations should be noted. First, NT signal enhancement reflects tracer and fluid movement both into the NTs and toward downstream pathways, making it essential to consider how experimental conditions affect not only the cribriform plate and nasal turbinates but also the brain and extracranial lymphatics. The temporal dynamics are as important as peak signal levels; e.g. aged mice showed higher enhancement alongside prolonged accumulation without increased clearance compared to young mice. Relying solely on peak values could therefore misrepresent efflux efficiency, as these differences likely reflect age-related changes in upstream and downstream compartments. Likewise, when parameters such as pre- and post-maximum slopes are not significant, as in the aging and zinc sulfate cohorts, additional metrics are needed to interpret the results. Some factors also remain unresolved due to the spatial resolution limits of DCE-MRI, which may introduce partial volume effects in small structures.

Second, using a single GBCA concentration with different injection rates may result in variable tracer levels in the NTs, affecting signal enhancement and confounding the observed dynamics. Additionally, the exclusive use of male mice leaves potential sex differences unaddressed, as CSF production rates vary between sexes.^69^ Third, while IVIS-CT provides sensitive optical readouts, it may underestimate deeper tracer accumulation. Fourth, since variation within our aging and olfactory sensory neuron ablation experiments is large, whereas the numbers of animals in these groups are relatively low, larger cohorts could have improved the reliability of our results. Lastly, our study focused on acute tracer dynamics, and longitudinal studies are needed to clarify whether impaired nasal clearance predicts disease progression or responds to interventions. These limitations notwithstanding, our multi-modal approach, which combines DCE-MRI, light microscopy, and large-animal validation, strengthens confidence in the robustness and translational relevance of our findings.

Accordingly, therapeutic strategies aimed at restoring or augmenting nasal CSF-lymphatic clearance merit further investigation. Beyond enhancing nasal efflux, recent work has identified the cervical lymphatic network as a tractable target: modulation of deep and superficial cervical lymphatic vessels improved CSF drainage in murine models.^28,90^ Leveraging agents or devices that promote cervical lymphatic contractility or vessel permeability, alongside interventions to preserve olfactory sensory integrity, could offer a multifaceted approach to mitigate glymphatic dysfunction and its clinical consequences. Moreover, further optimization of DCE-MRI sequences and data analysis pipelines is needed to better capture CSF drainage from intracranial spaces to extracranial lymphatics with higher resolution, improved SNR, and quantitative assessment of contrast agent concentrations across compartments. Similarly, MRI and *in vivo* light microscopy studies in higher mammals are needed to help bridge the translational gap between mice and humans. Lastly, broader use of olfactory neuronal manipulations is required to better elucidate the relationship between nasal CSF drainage and glymphatic function.

## Supplementary Material

fcag308_Supplementary_Data

## Data Availability

All data is available from the corresponding author upon a reasonable request. R code for statistical analysis can be found here: https://github.com/kswanberg/Osv/tree/main/Shanbhag_et_al_Efflux_manus_kod
