# Supplementary material for: Functional aspects of cerebrospinal fluid drainage through the cribriform plate
Source: Brain Commun. 2026 Aug 11;8(6):fcag308. doi: 10.1093/braincomms/fcag308 (PMC13748756; doi:10.1093/braincomms/fcag308)
Supplement: Supplementary file 1 [file fcag308_supplementary_data.docx]

**Supplementary Figures**

**Supplementary Figure 1**

**Supplementary Figure 1: Noninvasive superficial lymph node imaging with in vivo imaging system combined with computed tomography (IVIS-CT).** **(A)** An illustration of the experimental timeline. After setting scan parameters, a baseline is measured before cisterna magna (CM) injection of Lumiprobe-Cy7.5 is used for visualizing tracer accumulation with IVIS-CT under ketamine-xylazine (K/X) anesthesia. **(B)** IVIS-CT setup and regions of interest (ROI) for signal acquisition, with left and right middle cerebral artery (L/R-MCA) and the nasal turbinates (NTs). These were used as focus planes for signal gathering across mouse head and neck region. **(C)** Signal enhancement during different timepoints after CM injection of tracer. White arrow denotes the voxel containing superficial cervical lymph node when focus plane is MCA. n = Three mice. K/X, ketamine-xylazine; red arrow denotes deep cervical lymph node; ROI, regions of interest. Created in BioRender. Jukkola, J. (2026) <https://BioRender.com/ugzacaw>

**Alt-text:** Cisterna magna injection of tracer is shown to accumulate into brain, nasal turbinates, and superficial and deep cervical lymph nodes. The tracer seems to accumulate in the superficial cervical lymph nodes sooner than in the deep cervical lymph nodes.

**Supplementary Figure 2**

**Supplementary Figure 2:** **Normalized nasal turbinate (NT) and olfactory bulb (OB) signal enhancement in young and old mice using dynamic contrast-enhanced magnetic resonance imaging and OB analysis for amyloid beta (Aβ_1-42_) -coinjection experiments. (A)** Normalized signal (arbitrary units, a.u.) enhancement (by enhanced voxel count from Fig. 4). Dashed grey line indicates the start of cisterna magna (CM) tracer injection. **(B, C)** Normalized slopes from experiment start to maximum signal enhancement, and from maximum signal enhancement to experiment end. **(D)** Normalized rate of change. **(E)** Normalized area under curve (AUC). **(F)** Final AUC values from the last timepoint of (E). **(G)** Representative images including the region of interest (ROI) (dashed line) for olfactory bulb (OB). Scale bar is 1 mm. **(H)** Signal enhancement of the OBs in young and old mice. **(I)** Representative images including the ROI (dashed line) for OB analysis from the last timepoint of the BSA-647 - Aβ_1-42_ -coinjection experiments. Scale bar is 2 mm. **(J, K)** Mean pixel intensities of NTs and OBs. **(L)** Normalization of mean NT pixel intensity by mean OB pixel intensity. For all time series data, ANOVA on mixed models (*p* <0.05 for normalized NT signal enhancement and cumulative NT enhancement, but *p* >0.05 for rate of change and OB signal enhancement), followed by post hoc t-test for pairwise comparisons for each timepoint with Benjamini-Hochberg correction were used, where * = *p* <0.05. For non-time series group comparisons in (B), (C), (H) and (I), Student’s t-test were used, whereas for (F) and (J), Wilcoxon rank-sum was used, where * = *p* <0.05, ns = not significant. Spheres and shaded areas in time series represent mean value for each group from all mice per group, and standard error of the mean, respectively. Spheres and whiskers in bar charts represent each mouse and standard error of the mean, respectively. **(A-F)** For both age groups, n = Six mice. **(G-J)** For control and treatment group, n = 10-12 mice.

**Alt-text:** signal enhancement dynamics in old and young mice normalized to enhancing voxel numbers. Also, signal enhancement in the olfactory bulbs in both groups, without apparent differences in the dynamics. Finally, analysis for tracer and amyloid beta_1-42_ coinjection experiment regarding the olfactory bulbs, the nasal turbinates, and their comparative signal levels.

**Supplementary Figure 3**

**Supplementary Figure 3: Characterization of zinc sulfate’s** **(ZnSO_4_) effect on nasal turbinate (NT) tissue and normalized data for NT signal enhancement.** **(A-C)** Hematoxylin and eosin staining of nasal tissue close to cribriform plate (CP), with olfactory bulbs (OB), olfactory epithelium (OE), and submucosa (SM) at day 7 (both control = cntrl, and ZnSO_4_) and day 28 (ZnSO_4_). n = One mouse per group. Scale bars: 200 μm and 20 μm for the insets, respectively. **(D)** Scanning electron micrograph (SEM) of the NTs, with inserts from the main arches showing olfactory sensory neurons (OSN) at day 7. n = Three mice per group. Scale bars for SEM are 1 mm and 20 μm for the insets, respectively. **(E, H)** Dynamic contrast enhanced magnetic resonance imaging showing number of enhancing voxels at day 7 (E) and day 28 (H). **(F, I)** Normalized signal (arbitrary units, a.u.) enhancement data for both timepoints (by enhanced voxel counts from E and H, respectively). Dashed grey line indicates the start of cisterna magna injection. **(G, J)** Normalized rate of change data from both timepoints. For all time series data, ANOVA on mixed models (*p* <0.05 for normalized NT enhancement on day 28, but *p* >0.05) for other), followed by post hoc t-test for pairwise comparisons for each timepoint with Benjamini-Hochberg correction were used, where * = *p* <0.05. For non-time series group comparisons, Student’s t-test were used, where * = *p* <0.05, ** = *p* <0.01, ns = not significant. Spheres and shaded areas in time series represent mean value for each group from all mice per group, and standard error of the mean, respectively. Spheres and whiskers in bar charts represent each mouse and standard error of the mean, respectively. n = Four and five mice per group for day 7 and day 28, respectively.

**Alt-text:** Visual examination of olfactory sensory neuron ablation after seven and 28 days after intranasal administration of zinc sulfate. Additionally, cerebrospinal fluid contrast agent signal enhancement dynamics normalized to enhancing nasal turbinate voxel numbers in both seven and 28 day -groups.
